# Socio-Demographic and Lifestyle-Related Characteristics Associated with Self-Reported Any, Daily and Occasional Smoking during Pregnancy

**DOI:** 10.1371/journal.pone.0074197

**Published:** 2013-09-03

**Authors:** Ruth Baron, Judith Manniën, Ank de Jonge, Martijn W. Heymans, Trudy Klomp, Eileen K. Hutton, Johannes Brug

**Affiliations:** 1 Department of Midwifery Science, AVAG/EMGO Institute for Health and Care Research, Free University Medical Center, Amsterdam, The Netherlands; 2 Department of Midwifery Science, AVAG/EMGO Institute for Health and Care Research, VU University Medical Centre, Amsterdam, The Netherlands; 3 Department of Epidemiology and Biostatistics, VU University Medical Centre, Amsterdam, The Netherlands; 4 Department of Methodology and Applied Biostatistics, Faculty of Earth and Life Sciences, VU University Amsterdam, Amsterdam, The Netherlands; 5 Faculty of Health Sciences, McMaster University, Hamilton, Canada; 6 EMGO Institute for Health and Care Research, VU University Medical Centre, Amsterdam, The Netherlands; University of Liverpool, United Kingdom

## Abstract

Smoking during pregnancy is a risk factor for various adverse birth outcomes. In order to develop effective interventions, insight is needed into the characteristics associated with pregnant women who smoke. Unknown is whether these characteristics differ for women who smoke daily and women who smoke occasionally. Our study sample, drawn from the DELIVER study (Sept 2009-March 2011), consisted of 6107 pregnant women in primary care in the Netherlands who were up to 34 weeks pregnant. The associations of thirteen socio-demographic or lifestyle-related characteristics with ‘any smoking’, ‘daily smoking’ and ‘occasional smoking’ during pregnancy were tested using multiple binary logistic regression with general estimating equations (GEE). Characteristics most strongly associated with any smoking were low education (OR 10.3; 95% confidence interval (CI) 7.0-15.4), being of Turkish ethnicity (OR 3.9; 95%CI 2.3-6.7) and having no partner (OR 3.7; 95%CI 2.3-6.0). Women of Dutch ethnicity were three times more likely to smoke than those from Dutch-speaking Caribbean countries and non-religious women were much more likely to smoke than religious women. Low education was markedly more strongly associated with daily smoking than with occasional smoking (OR 20.3; 95%CI 13.2-31.3 versus OR 6.0; 95%CI 3.4-10.5). Daily smokers were more likely to be associated with other unfavorable lifestyle-related characteristics, such as not taking folic acid, being underweight, and having had an unplanned pregnancy. There is still much potential for health gain with respect to smoking during pregnancy in the Netherlands. Daily and occasional smokers appear to differ in characteristics, and therefore possibly require different interventions.

## Introduction

Maternal smoking is associated with higher risks of adverse birth outcomes such as preterm births [1], being small for gestational age (SGA) [2], intrauterine growth restriction [3], congenital heart defects [4] and stillbirth [5]. Maternal smoking is also associated with ill health later in childhood and throughout the life course, such as attention deficit hyperactivity disorder (ADHD) [6], childhood obesity [7], asthma [8], and tobacco dependence [9]. Although most smoking women who want to become or who are pregnant, do attempt to quit smoking, many of them continue to smoke during pregnancy [10].

An advisory committee was set up in 2009 for the Ministry of Health in the Netherlands to give recommendations for reducing adverse perinatal outcomes by reviewing relevant studies and consulting experts. Their report mentioned the prevention of smoking during pregnancy to be an important area for improving perinatal health [11]. Many other countries also have national goals for smoking cessation during pregnancy, such as the United States [12] and the United Kingdom [13]. A better understanding of socio-demographic and lifestyle-related characteristics associated with pregnant smokers will help to identify specific target groups for anti-smoking efforts among pregnant women and those who want to become pregnant. Various studies have measured the effects of different degrees of smoking during pregnancy and found dose–response associations between smoking and adverse outcomes [3,14,15]. Few studies, however, have differentiated between different degrees of smoking when investigating the characteristics associated with pregnant women who smoke. It is possible that there are differences between those who smoke daily and those who smoke occasionally [16] and that both groups may require different smoking cessation interventions.

The first research objective of this study was to identify the socio-demographic and lifestyle-related characteristics most strongly associated with any smoking during pregnancy in the Netherlands. The second objective was to assess any differences between the characteristics associated with self-reported daily and self-reported occasional smoking during pregnancy.

## Methods

### Study population

We obtained data from a dynamic prospective cohort study of 7865 pregnant women starting out in primary care from September 2009 to March 2011. These data constitute part of the DELIVER study and details about the design were published elsewhere [17]. Twenty midwifery practices distributed throughout the twelve provinces of The Netherlands were selected by means of purposive sampling, using region (north, east, south, west), level of urbanisation (urban or rural area) and practice type (dual or group) as stratification criteria. These midwifery practices invited all clients to participate by completing up to three questionnaires, which were either online or written, depending on their preference. Eligible clients were those who understood Dutch, English, Turkish or Arabic. The first questionnaire had to be completed at any point up to 34 weeks of pregnancy, the second between 35 weeks of pregnancy and giving birth, and the third after giving birth. Each practice invited clients during a period of twelve months. To improve overall response, written reminders were sent to all non-responders. Research assistants (student midwives) made telephone calls to all non-responders who had not responded within one week. Respondents of Turkish or Arabic ethnicity, who had not responded to the initial invitation were offered completion of the interviews by telephone in Turkish, Arabic, Berber or Dutch, depending on their preference. The overall net response was 62%. Ethical approval for this study was obtained from the Medical Ethics Committee of the VU University Medical Centre in Amsterdam.

### Study measures

For this study, we used data from the first questionnaire, which had been completed by women who were up to 34 weeks pregnant. Data on smoking, socio-demographic and lifestyle-related variables were used for the analyses. We selected thirteen independent variables based on prior literature which indicated a relationship with smoking during pregnancy [18–26].

Smoking: Respondents were asked whether they smoked daily, occasionally, or not at all. Three dichotomous dependent variables were formed distinguishing any smoking, daily smoking and occasional smoking from non-smoking.

Socio-demographic variables: Respondents reported their date of birth; age was subsequently categorized into 16-25 years, 26-35 years (reference category), and 36 years and older. Respondents reported the highest level of education they had completed, which was recoded into low (no education, only primary education or lower vocational education), medium education (only secondary school education or medium vocational education) and high education (college, university or post-graduate education; reference category). Neighbourhood social economic status (SES) was based on a ranking of postal codes, as was developed by the National Institute for Social Research (2006). This was based on average income, employment status and level of education per postal code area. We categorized below the 25^th^ percentile as low, between the 25^th^ and the 75^th^ percentile as medium and above the 75^th^ percentile as high. Respondents were asked about the country of birth of both parents. Ethnicity was based on the definition used by Statistics Netherlands, which considers someone to be of non-Dutch ethnicity if at least one of the parents was born in a country other than the Netherlands. If the parents were born in two different countries, then the mother’s country of birth is considered the ‘country of origin’. Distinctions were made between Dutch ethnicity (reference category) and the largest minority groups in the Netherlands, i.e. ‘Moroccan’, ‘Turkish’, ‘Dutch-speaking Caribbean’ (Surinamese /Antillean/Aruban) and ‘other ethnicities’. The respondents were also asked if they identified with a religious group or ideology, and if so, which one. Religion was then divided into five categories: ‘none (also including ‘not applicable’, ‘don’t know’ and ‘don’t wish to say’) (reference category), ‘Roman Catholic’, ‘Protestant’, ‘Islam’, and ‘Other religions’. Relationship status was based on a question asking the respondents whether or not they had a partner or spouse (‘yes’ was the reference category). Finally, respondents reported their number of children, which was then categorized into ‘no children’ (reference category), ‘1-2 children’, ‘3-4 children’ and ‘5 children or more’.

Lifestyle-related variables: respondents were asked whether or not they had consumed any alcohol since knowing they were pregnant, with answer options ‘yes’ or ‘no’ (‘no’ is reference category). The variable Body Mass Index (BMI) was based on self-reported height and weight before the start of pregnancy and coded into ‘underweight’ (<18.5 kg/m^2^), normal weight (18.5-24.99 kg/m^2^; reference category), overweight (25-29.99 kg/m^2^) and obese (≥30kg/m^2^), according to the World Health Organization classification of BMI. Folic acid supplementation was based on the question of whether respondents were taking/had taken folic acid during this pregnancy, with response options ‘yes’ (reference category) and ‘no’. Respondents were asked about their current mood and had three response options ‘not at all anxious or depressed’ (reference category), ‘somewhat anxious or depressed’ and ‘very anxious or depressed’. Respondents were also asked whether their present pregnancy was planned (reference category) or unplanned. Finally, the respondents were asked to which extent they believed they could influence their health by their own behaviours on a four point scale, which was dichotomized into ‘hardly any/no control’ and ‘quite a bit/very much control’ (reference category).

### Statistical analyses

We obtained descriptive statistics by running frequencies, percentages and means. Preliminary analyses of missing data showed that there were 8.6% cases with missing data and most variables contained less than 1% of missing data each. The variable BMI, based on the variables height and weight contained 6.2% missing data. Logistic regression with response versus non-response as outcome showed several characteristics to be strongly associated with non-response of BMI, including low education and ethnic minority. This was a strong reason for assuming it to be of the type ´missing at random´ (MAR) and that it was therefore suitable for carrying out an imputation technique. We considered the use of multiple imputation, which leads to the pooled results of multiple newly generated datasets. However, there is currently not a way of obtaining an overall p-value for categorical variables in SPSS, which is necessary for selective backward logistic regression. Due to this limitation and the fact that there were few missing values in the variables, we chose to carry out single stochastic regression imputation based on the Multivariate Imputation by Chained Equation (MICE) algorithm.

As the respondents in our study population were clustered within twenty different midwife practices, it is possible that their responses were not completely independent, but that there were correlations of responses within each practice. We accounted for this potential dependency of measurements in each practice using generalized estimating equations (GEE). Univariable and multivariable relationships of the thirteen independent variables with the dependent variable ‘any smoking’ were assessed using GEE for backward binary logistic regression. In a stepwise fashion, the least significant characteristic was removed from the GEE model and logistic regression performed again. This procedure was repeated until only significant characteristics (p<0.05) remained in the model. Confidence intervals of odds ratios were set at 95%.

Secondary analyses were then carried out to assess the characteristics associated with self-reported daily smoking (versus no smoking) and occasional smoking (versus no smoking). We performed GEE for multivariable backward logistic regression with all thirteen independent variables using the same procedure described above for ´any smoking´. As the number of cases in the dependent variables ‘daily smoking’ and ‘occasional smoking’ were smaller in the secondary analyses, it was necessary to reduce the number of categories in the independent variables. Although there were differences between the different religions when examining their relationship with ‘any smoking’, all religions showed a trend in the same direction. We decided therefore to categorize the variable religion into ‘no religion’(reference) versus ‘any religion’ for the ‘daily smoking’ and ‘occasional smoking’ models. The odds ratios and confidence intervals obtained from the GEE models were used for presentation. All analyses were performed in IBM SPSS version 20.

## Results

In total, 6107 women completed the first questionnaire of the DELIVER study. Maternal baseline characteristics are presented in table 1. Median gestational age was 19 weeks; 9.2% of respondents were smokers (5.4% daily and 3.8% occasional). Daily smokers reported smoking 7.8 (SD 4.4, range 1.5 to 20) cigarettes per day on average and occasional smokers 8.8 (SD 8.1, range 0-50) on average per week (i.e. 1.2 per day).

**Table 1:** Baseline characteristics of the study population (n=6107).

**Baseline characteristics**		N (%)*
**Smoker status**	-Non-smokers	5522 (90.8%)
	-Total smokers	562 (9.2%)
	*-Daily/mean per day(SD)*	331 (5.4%) / 7.8 (4.4)
	*-Occasional/mean per wk(SD)*	231 (3.8%) / 8.8 (8.1)
	-Missings	23
**Gestational age (wks)**	-Median (P5:P95)	19 (11:32)
	-Missings	31
**Age**	-Mean(SD)	30.4 (4.6)
	-Range	16-48
	-26-35	4422 (72.5%)
	-16-25	864 (14.2%)
	-36+	814 (13.3%)
	-Missings	7
**Education**	-High	2971 (48.8%)
	-Medium	2190 (36.0%)
	-Low	923 (15.2%)
	-Missings	23
**Neighbourhood SES**	-High	1493 (24.6%)
	-Medium	2940 (48.3%)
	-Low	1648 (27.1%)
	-Missings	26
**Ethnicity**	-Dutch	5092 (83.6%)
	-Surinamese/Antillian/Aruban	91 (1.5%)
	-Moroccan	135 (2.2%)
	-Turkish	111 (1.8%)
	-Other	659 (10.8%)
	-Missings	19
**Relationship status**	-Spouse/Partner	5993 (98.3%)
	-No spouse/partner	101 (1.7%)
	-Missings	13
**Religion**	-None/N.A./don’t know/don’t wish to say	3646 (60.1%)
	-Roman Catholic	632 (10.4%)
	-Protestant	1412 (23.3%)
	-Islamic	318 (5.2%)
	-Other religions	55 (0.9%)
	-Missings	44
**No. children**	-0	2762 (45.3%)
	-1-2	3079 (50.5%)
	-3-4	230 (3.8%)
	-5+	30 (0.5%)
	-Missings	6
**Pregnancy**	-Planned	5018 (82.3%)
	-Unplanned	1076 (17.7%)
	-Missing	13
**Folic acid supplementation**	-Yes	5560 (91.3%)
	-No	527 (8.7%)
	-Missings	20
**Alcohol (at least 1x)**	-No	5408 (89.0%)
	-Yes	668 (11.0%)
	-Missings	31
**BMI**	-Normal	3837 (67.1%)
	-Underweight	194 (3.4%)
	-Overweight	1247 (21.8%)
	-Obese	441 (7.7%)
	-Missings	388
**Depressed/ anxious mood**	-Not at all	4871 (80.0%)
	-A little	1165 (19.1%)
	-Very much	49 (0.8%)
	-Missings	22
**Health control belief**	-Quite a bit to very much	5152 (84.8%)
	-Hardly any to none	925 (15.2%)
	-Missings	30

- Frequencies and percentages are based on the original dataset, excluding the missing values

- Results are presented as N(%), unless stated otherwise

- N.A. = Not applicable

### Any smoking during pregnancy

Multivariable analyses showed that eleven of the thirteen variables were significantly associated with smoking during pregnancy (Table 2). Education was the strongest characteristic with women of low education being more than ten times more likely to smoke than women of high education. Those who had no religion were five times more likely to smoke than those who were Islamic, 2.5 times more likely than those who belonging to a Protestant church and 1.5 times more likely to smoke than those who were Roman Catholic. Women of Turkish ethnicity were almost four times more likely to smoke than women of Dutch ethnicity. Those with Dutch-speaking Caribbean ethnicities (Surinamese/ Antillean or Aruban) were three times less likely to smoke than those of Dutch ethnicity. Those who had no partner or spouse were more than three times more likely to smoke than those with a partner or spouse. Other characteristics with smaller effects, but still significantly associated with smoking, were being underweight, current depressed or anxious mood, low neighbourhood SES, not taking folic acid, alcohol consumption at least once during pregnancy, unplanned pregnancy and low health control beliefs.

**Table 2:** Odds ratios (OR) and 95% confidence intervals (95% CI) showing the results of univariable and multivariable backward logistic regression analyses (GEE models), testing associations of socio-demographic and lifestyle-related variables with any, daily and occasional smoking during pregnancy.

**Socio-demographic & lifestyle-related variables**		**Any smoking during pregnancy**			**Daily smoking**		**Occasional smoking**	
		**No. of smokers (%)**	**Univariable OR(95%CI)**	**Multivariable OR(95%CI)**	**No. of smokers (%)**	**Multivariable OR(95%CI)**	**No. of smokers (%)**	**Multivariable OR(95%CI)**
**Age**	-26-35	336/4426 (7.6)	1		186/4276 (4.3)		150/4240 (3.5)	
	-16-25	165/865 (19.1)	**2.8 (2.1-3.7)**		110/810 (13.6)		55/755 (7.3)	
	-36+	64/816 (7.8)	1.1 (0.9-1.3)		38/790 (4.8)		26/778 (3.3)	
**Education**	-High	78/2981 (2.6)	1	1	30/2933 (1.0)	1	48/2951 (1.6)	1
	-Medium	251/2198 (11.4)	**4.7 (3.4-6.5)**	**4.5 (3.2-6.3)**	143/2090 (6.8)	**7.1 (4.6-10.8)**	108/2055 (5.3)	**3.2 (2.1-5.0)**
	-Low	236/928 (25.4)	**12.4 (8.3-18.6)**	**10.3 (7.0-15.4)**	161/853 (18.9)	**20.3 (13.2-31.3)**	75/767 (9.8)	**6.0 (3.4-10.5)**
**Neighbourhood SES**	-High	100/1497 (6.7)	1	1	57/1454 (3.9)	1	43/1440 (3.0)	1
	-Medium	251/2953 (8.5)	1.1 (0.9-1.5)	1.1 (0.8-1.4)	149/2851 (5.2)	1.1 (0.8-1.5)	102/2804 (3.6)	1.0 (0.7-1.5)
	-Low	214/1657 (12.9)	**1.9 (1.5-2.2)**	**1.5 (1.3-1.8)**	128/1571 (8.1)	**1.5 (1.2-1.9)**	86/1529 (5.6)	1.4 (1.0-2.0)*
**Ethnicity**	-Dutch	464/5108 (9.1)	1	1	282/4926 (5.7)	1	182/4826 (3.8)	1
	-Surinamese/Antill/Aruban	6/91 (6.6)	0.8 (0.4-1.5)	**0.3 (0.1-0.8)**	4/89 (4.5)	0.3 (0.1-1.0)*	2/87 (2.3)	0.4 (0.1-1.3)
	-Moroccan	8/137 (5.8)	0.7 (0.3-1.4)	0.6 (0.2-1.4)	5/134 (3.7)	**0.3 (0.1-0.9)**	3/132 (2.3)	0.5 (0.2-1.6)
	-Turkish	27/112 (24.1)	**3.2 (2.2-4.7)**	**3.9 (2.3-6.7)**	13/98 (13.3)	**1.9 (1.2-3.0)**	14/99 (14.1)	**3.4 (2.2-5.4)**
	-Other	60/659 (9.1)	1.1 (0.8-1.4)	0.9 (0.7-1.3)	30/629 (4.8)	0.7 (0.5-1.2)	30/629 (4.8)	1.3 (0.9-1.7)
**Relationship status**	-Spouse/Partner	527/6006 (8.8)	1	1	304/5783 (5.3)	1	223/5702 (3.9)	
	-No spouse/partner	38/101 (37.6)	**6.2 (4.3-8.9)**	**3.7 (2.3-6.0)**	30/93 (32.3)	**4.9 (3.1-7.9)**	8/71 (11.3)	
**Religion**	-None/N.A./don’t know/ don’t wish to say	404/3678 (11.0)	1	1	249/3523 (7.1)	1	155/3429 (4.5)	1
	-Roman Catholic	51/633 (8.1)	**0.7 (0.5-0.9)**	**0.6 (0.5-0.9)**	85/2353 (3.6)**	0.4 (0.3-0.5)**	76/2344 (3.2)**	0.6 (0.4-0.8)**
	-Protestant	72/1421 (5.1)	**0.4 (0.3-0.5)**	**0.4 (0.3-0.5)**				
	-Islamic	36/320 (11.2)	1.0 (0.7-1.5)	**0.2 (0.1-0.4)**				
	-Other religions	2/55 (3.6)	0.4 (0.1-1.1)	0.3 (0.1-1.4)				
**No. of children**	-0	267/2765 (9.7)	1		161/2659 (6.1)		106/2604 (4.1)	
	-1-2	271/3081 (8.8)	0.9 (0.8-1.1)		158/2968 (5.3)		113/2923 (3.9)	
	-3-4	26/231 (11.3)	1.2 (0.8-1.7)		15/220 (6.8)		11/216 (5.1)	
	-5+	1/30 (3.3)	0.3 (0.0-2.6)		0		1/30 (3.3)	
**Pregnancy**	-Planned	402/5027 (8.0)	1	1	227/4852 (4.7)	1	175/4800 (3.6)	
	-Unplanned	163/1080 (15.1)	**2.1 (1.7-2.5)**	**1.4 (1.1-1.8)**	107/1024 (10.4)	**1.5 (1.1-2.0)**	56/973 (5.8)	
**Folic acid supplement**	-Yes	470/5578 (8.4)	1	1	268/5376 (5.0)	1	202/5310 (3.8)	
	-No	95/529 (18.0)	**2.3 (1.8-3.1)**	**1.5 (1.1-1.9)**	66/500 (13.2)	**1.6 (1.1-2.3)**	29/463 (6.3)	
**Alcohol (at least 1x)**	-No	498/5439 (9.2)	1	1	293/5234 (5.6)	1	205/5146 (4.0)	1
	-Yes	67/668 (10.0)	1.2 (0.9-1.6)	**1.4 (1.1-1.9)**	41/642 (6.4)	**1.6 (1.1-2.3)**	26/627 (4.1)	1.4 (1.0-1.8)*
**BMI**	-Normal	339/4053 (8.4)	1	1	202/3916 (5.2)	1	137/3851 (3.6)	
	-Underweight	49/229 (21.4)	**2.9 (2.2-3.8)**	**2.1 (1.6-2.8)**	35/215 (16.3)	**2.6 (1.8-3.7)**	14/194 (7.2)	
	-Overweight	136/1356 (10.0)	1.2 (0.9-1.5)	1.0 (0.8-1.3)	77/1297 (5.9)	0.9 (0.7-1.2)	59/1279 (4.6)	
	-Obese	41/469 (8.7)	1.0 (0.7-1.4)	0.7 (0.5-1.1)	20/448 (4.5)	**0.6 (0.3-0.9)**	21/449 (4.7)	
**Depressed/anxious mood**	-Not at all	397/4886 (8.1)	1	1	232/4721 (4.9)		165/4654 (3.5)	1
	-A little	157/1171 (13.4)	**1.7 (1.5-2.0)**	**1.3 (1.1-1.6)**	96/1110 (8.6)		61/1075 (5.7)	1.3 (1.0-1.8)*
	-Very much	11/50 (22.0)	**3.2 (2.0-5.0)**	**1.8 (1.1-2.9)**	6/45 (13.3)		5/44 (11.4)	**2.7 (1.2-5.8)**
**Health control belief**	-Quite a bit/very much	397/5178 (7.7)	1	1	231/5012 (4.6)	1	166/4947 (3.4)	
	-Hardly any to none	168/929 (18.1)	**2.6 (2.1-3.3)**	**1.4 (1.1-1.7)**	103/864 (11.9)	1.3 (1.0-1.6)*	65/826 (7.9)	

Odds Ratios in **Bold** Are significant

*Rounding Error : p<0.05

**All Religions

N.A. = Not Applicable

### Daily smokers versus non smokers

Education was the characteristic most strongly associated with daily smoking, with pregnant women of low education being twenty times more likely to be daily smokers compared to those of high education. Having no partner, being of Turkish or Dutch ethnicity, having no religion and being underweight also appeared to be characteristics strongly associated with daily smoking. Other characteristics with smaller effects, but still significantly associated with daily smoking, were alcohol consumption at least once during pregnancy, not taking folic acid, unplanned pregnancy, low neighbourhood SES and low health control beliefs.

### Occasional smokers versus non smokers

Education was also the strongest characteristic associated with occasional smoking, but with less extreme odds; women of low education were six times more likely to smoke occasionally than those of high education. Other characteristics which were quite strongly associated with occasional smoking were Turkish ethnicity and current depressed or anxious mood. Other characteristics with smaller effects, but still significantly associated with occasional smoking were not identifying with a religion, low neighbourhood SES and alcohol consumption at least once during pregnancy.

## Discussion

### Any smoking during pregnancy

Our study showed that 9.2% of pregnant women reported smoking during pregnancy. Due to the fact that higher educated women were over-represented in our study population (48.8% versus 26.7% in the general Dutch population of women of 15-65 years [27]), the true prevalence of smoking in the Dutch population of pregnant women is likely to be considerably higher.

Eleven out of the thirteen characteristics tested for their association with smoking were statistically significant, implying that there is a range of factors associated with smoking during pregnancy. The widest disparity is seen between low and high education with respect to ‘any smoking’ (25.2% versus 2.6%), which is in line with the results of earlier studies in the Netherlands [28]. Although there is evidence that this educational gap may be narrowing [29], our study shows that this gap is still substantial and should be a main target for intervention.

Our results also revealed that religious and ethnic background and having a partner may be relevant issues to take into account in smoking cessation interventions. Pregnant women who were not religious were more likely to smoke than those with a religion, with those of Islamic religion being the least likely to smoke. Studies from other countries have also shown that women who report being religious are less likely to smoke during pregnancy [20,30]. Some elements of main stream religions include a focus on avoiding unhealthy habits, and attendance of organized services and social events, the latter of which may be a good foundation of social support and care [31].

Having no partner was also strongly associated with smoking in our study. A study by Siahpush [24] found that single mothers who smoked were more likely to have financial deficiencies, to be depressed or anxious, to be living in disadvantaged areas, to be surrounded by many other smokers, and to have smoked since an early age. A partner may also be a good source of social support and care. A study by Elsenbruch et al. [32] measured different levels of perceived social support in pregnant women and characteristics associated with these levels. Those with low social support were more likely to have an unplanned pregnancy, to not have a partner, be younger, less educated, depressed and to smoke. In our study most of these factors identified with low social support were associated with smoking, implying that low social support could possibly be an underlying factor to consider when designing interventions.

Being of Turkish ethnicity was strongly associated with smoking during pregnancy, when compared to all other ethnicities. Dutch ethnicity was strongly associated with smoking compared to the Dutch-speaking Caribbean ethnicities. Both Turkey and the Netherlands have high rates of smoking in their general populations [33] suggesting that an approach involving other smoking family members besides the pregnant women in smoking cessation programs may be beneficial. Most smoking cessation programs focus only on pregnant women [34] but studies have shown that women whose partners had quit smoking were much more likely to quit themselves [35].

Although the odds were less high, other characteristics which are risk factors in themselves for adverse pregnancy outcomes, such as not taking folic acid and having consumed alcohol at least once, were also significantly associated with smoking. Haslam and Lawrence [21] also found that pregnant smokers were less likely to take folic acid supplementation. El Marroun et al. [18] from the Generation R study [36] found that women who used cannabis were also more likely to use tobacco and alcohol. It is likely that certain social groups have multiple unfavourable lifestyle-related risk factors for adverse birth outcomes, which may be an important issue when it comes to developing smoking cessation interventions.

### Characteristics of daily smoking versus occasional smoking

Self-reported daily smokers did not necessarily all smoke more cigarettes per week than self-reported occasional smokers (7.8 cigarettes per day versus 8.8 per week). Although dose–response relationships have been shown between smoking and adverse outcomes [3,14,15], it is unclear how timing between cigarettes influences adverse outcomes, and whether occasional ‘binge’ smoking has the same effects as daily smoking. Although they may both be equally at risk for having adverse birth outcomes, there appear to be differences in characteristics between the groups. The difference in odds for smoking between lower and higher education was markedly larger for daily smoking (lower educated women were twenty times more likely to smoke) than for occasional smoking (lower educated women were almost six times more likely to smoke). Daily smoking was also associated with more lifestyle-related factors than occasional smoking, such as unplanned pregnancy, not taking folic acid and being underweight. Erickson and Arbour [37] showed that heavy smoking was more likely to be associated with other risk factors including drug and alcohol use, fewer prenatal care visits and being a single parent, leading the authors to suggest heavy smoking during pregnancy could be used as a marker for other risk factors.

In a study by Haight et al. [16] amongst college student smokers, it was suggested that daily smokers are driven by internal cues (ie. physical addiction) and occasional smokers by external cues (ie. pressure from social activities). It is plausible that these differences are also present within pregnant women, which implies that these two groups may require different interventions. Daily smokers may benefit more from approaches focused on diminishing the physical and psychological addiction to smoking. As they are also more likely to be faced with multiple other health risks, they may benefit from interventions focused on general health promotion. Encouraging folic acid supplementation for example, may not only decrease the risk of neural tube defects, but may even reduce some of the negative adverse outcomes associated with smoking during pregnancy [38]. Interventions for occasional smokers may need to focus on how to resist and cope with external cues, such as going to events where friends and family members are smoking.

### Limitations

The women in this study filled in the questionnaire at different points up to 34 weeks of pregnancy. This study, therefore, does not take into account that some of these women may have stopped smoking just before or after participating in this study. Our large study does provide a good cross-sectional insight into the characteristics associated with women who are smoking at various gestational ages.

Our study relied on self-reported smoking status and smoking was not objectively validated by means of carbon monoxide (CO) measurements. It is therefore possible that smoking was underreported in our study, as other studies have found as well [39,40]. This is likely to be minimal, however, as the respondents were informed that their details would remain anonymous to everyone, including their midwives.

We also had to rely on the self-perception of women in whether they considered themselves daily or occasional smokers; it is possible therefore that there was variation in how women interpreted the term ‘occasional’. However, daily smokers subsequently had to report how many cigarettes they smoked daily and occasional smokers how many cigarettes they smoked weekly. The average difference in cigarettes smoked per day in the two groups (7.8 versus 1.2) suggests that daily and occasional smokers do actually have different smoking behaviours.

Our study population also consisted of relatively more highly educated women than in the general Dutch population, making the prevalence of smoking found in our study likely to be an underestimation of the prevalence in the general population. Furthermore, our study consisted of women starting pregnancy in primary care and who were therefore considered low risk. In the Netherlands, about 83.9% of all pregnant women start their pregnancy in primary care. The remaining 16.1% of women start their pregnancy in secondary care, generally due to obstetrical high risk factors [41]. Women starting in primary care differ from those starting in secondary care with regard to several socio-demographic characteristics. They are more likely to be under 35 years old, have a higher socioeconomic status and a Western ethnicity [42]. This study can therefore be considered to be representative of the majority of women living in the Netherlands, but cannot be extrapolated to the population of women at high obstetric risk. However, this study aimed to show the characteristics associated with smoking during pregnancy, and we have no reason to believe that these associations are likely to be very different based on the type of prenatal care.

## Conclusions

These results show that there is still much potential for health gain with respect to smoking during pregnancy in the Netherlands. The characteristics most strongly associated with any smoking during pregnancy were low education, not being religious, being of Turkish or Dutch ethnicity and having no partner. Awareness of these groups at risk and consideration of possible underlying factors such as lack of social support, is necessary for designing effective anti-smoking cessation programs. Daily smokers and occasional smokers appear to differ with respect to socio-demographic and lifestyle-related characteristics, implying that they may require different interventions.
